# A SYSTEMATIC REVIEW OF DIGITAL INTERVENTIONS ADDRESSING MENTAL DISORDERS AMONG THE ELDERLY POPULATION

**DOI:** 10.1093/geroni/igz038.1895

**Published:** 2019-11-08

**Authors:** Md Mahbub Hossain, Samia Tasnim, Abida Sultana, Nishat Tasnim Hasan, Hoimonty Mazumder, Nusrat Khan, Farah Faizah

**Affiliations:** 1 Texas A&M School of Public Health, College Station, Texas, United States; 2 Nature Study Society of Bangladesh, Khulna, Bangladesh; 3 Independent Researcher, College Station, Texas, United States; 4 University of Cambridge, Cambridge, United Kingdom; 5 United Nations Population Fund (UNFPA), Dhaka, Bangladesh

## Abstract

Background: Many studies have reported the widespread application of digital technologies in improving mental health. However, little is known about how these technological advancements can help the geriatric population who suffer from a wide range of mental disorders. There is no extensive review of evidence which can guide effective policy-making and implementation of such interventions. Objectives: To identify digital interventions addressing mental disorders among elderly people and evaluate the outcomes of these interventions. Methodology: According to the PRISMA guidelines, we searched six major health databases and screened the literature using these criteria: 1) journal articles reporting an intervention delivered using any of the digital platforms, 2) the interventions aimed to improve at least one mental disorder among geriatric population, 3) articles published in English language, 4) studies conducted in in any settings and time frame reporting any of the mental health-related outcomes. Results: Among 4870 articles found in the preliminary literature search, only 19 studies met our criteria. Most of the studies (n=14) described digital interventions addressing depressive illness among the elderly population. However, many interventions targeted multiple mental conditions including dementia, stress, anxiety, mood disorders, phobia, and functional disabilities. These interventions used internet-based therapies, mindfulness, digital assistants, and applications improving mental health behavior and practices. Most of the interventions (n=12) were evaluated using randomized study designs. Reported outcomes included improved symptoms, better quality of living, emotional and functional advancements, and decreased cost of treatment. This evidence necessitates further research and application of such technologies to improve geriatric mental health.

